# Molecular typing of stage IB non-small-cell lung cancer for precision medicine

**DOI:** 10.3389/fonc.2026.1605054

**Published:** 2026-03-13

**Authors:** Xiaoyan Li, Zitong Wan, Miaomiao Wen, Jianfei Zhu, Kaiqi Wei, Xiaohong Ji, Jiao Zhang, Guoyin Li, Jie Lei, Yangbo Feng, Yanlu Xiong, Yinxi Zhou, Shaowei Xin

**Affiliations:** 1 Department of Blood Transfusion, Shanxi Provincial People’s Hospital, Taiyuan, China; 2 Department of Thoracic Surgery, Tangdu Hospital, Fourth Military Medical University, Xi’an, China; 3 Department of Thoracic Surgery, Shaanxi Provincial People’s Hospital, Xi’an, China; 4 College of Life Science and Agronomy, Zhoukou Normal University, Zhoukou, China; 5 Academy of Medical Science, Zhengzhou University, Zhengzhou, China; 6 Innovation Center for Advanced Medicine, Tangdu Hospital, Fourth Military Medical University, Xi’an, China; 7 Department of Thoracic Surgery, The First Medical Center, Chinese People's Liberation Army (PLA) General Hospital, Beijing, China; 8 Department of Thoracic Surgery, Air Force Medical Center, People's Liberation Army (PLA), Beijing, China; 9 Department of Thoracic Surgery, 962 Hospital of the Joint Logistics Support Force, Harbin, Heilongjiang, China

**Keywords:** carboxypeptidase D, molecular typing, non-small-cell lung cancer, prognosis, stage IB

## Abstract

**Background:**

The optimal postoperative management of stage IB non-small-cell lung cancer (NSCLC) remains controversial due to significant heterogeneity. This study aimed to establish a molecular classification for stage IB NSCLC to guide precision medicine.

**Methods:**

We performed consensus clustering of transcriptomic profiles from TCGA-NSCLC (training set, n=119) and validated in GSE31210 (n=53). Molecular subtypes were compared with stage IA/II patients for clinical significance. Differentially expressed genes were analyzed using LASSO regression to identify biomarkers. Functional studies were conducted using patient-derived organoids (PDOs), cell lines, and tissue specimens.

**Results:**

Stage IB NSCLC was divided into two subtypes (IB1 and IB2) with significantly different disease-free survival (DFS). IB1 patients showed DFS similar to stage IA, while IB2 patients exhibited DFS comparable to stage II (especially IIB). Carboxypeptidase D (CPD) was identified as a key biomarker capable of distinguishing high- and low-risk patients (AUC=0.874 in validation). CPD was overexpressed in tumor tissues and promoted proliferation and migration in PDOs and cell lines.

**Conclusion:**

This molecular classification effectively stratifies stage IB NSCLC into low-risk (IB1, observation suitable) and high-risk (IB2, adjuvant therapy warranted) subgroups. CPD serves as a promising biomarker for clinical risk stratification.

## 1 Introduction

The optimal management of resectable stage IB non−small−cell lung cancer (NSCLC), particularly the indication for postoperative adjuvant therapy, remains controversial. A core tenet of precision medicine in this context lies in dissecting tumor heterogeneity—specifically, identifying high-risk and low-risk subtypes. Previous studies suggested stage IB patients with high-risk clinicopathological features, such as larger tumor size, solid/micropapillary components and visceral pleural invasion, exhibit poorer outcomes and may derive benefit from postoperative adjuvant chemotherapy (1–4). Emerging evidence suggests that adjuvant targeted therapy as well as adjuvant or neoadjuvant immunotherapy could improve outcomes in a subset of stage IB patients with specific genetic or immune statuses (5–7). Consistent with these advances, a proportion of stage IB NSCLC (tumors 4–5 cm) defined in the 7th edition staging system was reclassified as stage IIA in the 8th edition (8). The 2023 National Comprehensive Cancer Network (NCCN) guidelines also note that adjuvant chemotherapy may be considered for stage IB patients with high-risk factors, including poorly differentiated tumors (excluding well-differentiated neuroendocrine tumors), vascular invasion, wedge resection, and visceral pleural invasion (8).

Nevertheless, current risk stratification remains mostly grounded in macroscopic pathological characteristics, which offer limited discriminatory power. Molecular typing has the potential to substantially deepen our understanding of tumor heterogeneity and refine treatment strategies (9). Yet a dedicated molecular classification for stage IB NSCLC is still lacking. In this study, we performed, for the first time, molecular classification of stage IB NSCLC based on whole-transcriptome profiles, an approach with clear clinical implications. Furthermore, we identify a specific gene signature and investigate the biological role of key genes in the malignant progression of stage IB disease.

## 2 Materials and methods

### 2.1 Transcriptomic and clinical information

The RNA sequencing (RNA-seq) and biospecimen-derived clinical data of The Cancer Genome Atlas (TCGA)-lung adenocarcinoma (LUAD) and TCGA-lung squamous cell carcinoma (LUSC) were downloaded using the R package TCGA-assembler (10). Clinical data, especially prognostic information, were retrieved from TCGA Pan-Cancer Clinical Data Resource (TCGA-CDR) (11). The TCGA-NSCLC cohort was used as the training set. For training cohort, we obtained clinical data from 522 LUAD patients and 504 LUSC patients from TCGA, among whom 270 LUAD patients and 220 LUSC patients with a defined AJCC 7th edition staging classification. This subset included 119 stage IB patients (60 LUAD, 59 LUSC); none had received neoadjuvant therapy (with 110 normal tissue samples available). The transcriptome data and clinical information of GSE31210 and GSE30219 were downloaded by the R package GEOquery for validation (26–28). For the validating cohorts, transcriptome data and clinical information of GSE31210 (GPL570 platform, 53 stage IB patients and 20 normal tissues) and GSE30219 (GPL570 platform, 13 stage IB patients and 14 normal tissues) were further downloaded by the R package GEOquery. NSCLC patients (LUAD and LUSC subtypes, staged according to the 7th edition AJCC system, which can be converted to the 8th edition) were retrieved from the SEER Database using SEER*Stat software (version 8.3.9.2), including 6138 stage IB, 14836 stage IA, and 10125 stage II patients (5036 stage IIA, 5012 stage IIB) with available disease-specific survival (DSS) data (survival time > 1 month) (29).

### 2.2 NSCLC specimens and immunohistochemistry

A total of 10 stage IB NSCLC specimens (5 paired cancerous tissues and para-cancerous tissues) were collected from Tangdu Hospital, Fourth Military Medical University (Xi’an, China). Briefly, specimens were dewaxed, underwent antigen repaired, treated with hydrogen peroxide, blocked and incubated with specific primary antibodies (anti-Ki67 antibody: GB111499, Servicebio, Wuhan, China; anti-CPD antibody: DF9339, Affinity Biosciences, OH, USA) followed by horseradish peroxidase (HRP)- conjugated secondary antibody. Subsequently, the specimens were stained, counterstained, and observed under a microscope.

### 2.3 Cell lines

Human NSCLC cell lines (H1975 and H358), purchased from the Cell Bank of the Chinese Academy of Sciences (Shanghai, China) and authenticated by short tandem repeat (STR) profiling. These cells were cultured in RPMI−1640 supplemented with 10% fetal bovine serum (FBS) at 37˚C in a humidified atmosphere containing 5% CO2.

### 2.4 Transfection

In brief, siRNAs (RiboBio, Guangzhou, China) and plasmids (Hanbio; Sangon Biotech, Shanghai, China) were transfected into cells using Lipofectamine 2000 (Invitrogen, CA, USA) according to the manufacturer’s instructions. The siRNA sequences were as follows: CPD siRNA1: 5-GGAACAGAATCGAAGATCA-3; CPD siRNA2: 5-GAAGTCCTATCCATTTGTA-3.

### 2.5 Quantitative reverse transcription-polymerase chain reaction

Total RNA from patient-derived organoids (PDOs) and 30stage IB NSCLC cancerous tissues, collected from Tangdu Hospital, Fourth Military Medical University (Xi’an, China), was extracted using RNAiso reagent (TaKaRa, Dalian, China). cDNA was synthesized from 1.0 μg of total RNA using the PrimeScript RT Master Mix (TaKaRa). Real-time PCR was performed using SYBR Premix Ex Taq II (TaKaRa) on a Mx3005p Real-Time PCR detection system (Agilent Technologies, CA, USA). β-actin served as internal reference. The 2−ΔΔCt method was used to determine relative gene expression. CPD, forward: 5- ATGCCCATGGTAACCAGCAT-3 and

reverse: 5- ACTTTACTGCCATGCTTTAGACA-3;

β-actin, forward:

5-GATCATTGCTCCTCCTGAGC-3 and

reverse: 5-ACTCCTGCTTGCTGATCCAC-3.

### 2.6 Western blot

Proteins from cell lysates were separated by SDS-PAGE and transferred to a nitrocellulose membrane, which was incubated with primary specific antibodies (β-actin: 4970S, CST, MA, USA; CPD, PA5-103707, Invitrogen, MA, USA), followed by HRP-conjugated secondary antibody. ECL reagent (Merck Millipore, Darmstadt, Germany) was applied for protein detection.

### 2.7 Plate colony forming experiments

Cells in the logarithmic growth stage were fully mixed into a single cell suspension, seeded in six-well plates with 2 ml of medium containing 10% FBS. After 2–3 weeks, colonies were stained with crystal violet, and the colony area was quantified using Image-Pro Plus (Media Cybernetics, MD, USA). At least three independent experiments were performed in triplicate.

### 2.8 Transwell assay

Cells in the logarithmic growth stage were suspended in medium containing no FBS and seeded into the Transwell chamber (Corning, NY, USA), and culture medium containing 20% FBS was added to the 24-well plate, where the Transwell chambers were placed. After 24 hours of incubation, the cells were fixed in methanol and stained with crystal violet. And the area of cells resident on the lower surface of the filter was measured by Image-Pro Plus (Media Cybernetics). At least three independent experiments were performed in triplicate.

### 2.9 Patient-derived organoid culture

Briefly, fresh stage IB NSCLC tissues obtained from Tangdu Hospital, Fourth Military Medical University, were minced into pieces, washed and dissociated in digestion buffer (Advanced DMEM/F12 medium (Lonza, Basel, Switzerland) with 10% penicillin/streptomycin, 1.5 mg/mL collagenase type II, 500 U/mL collagenase type IV, 0.1 mg/mL dispase type II, 10 μM Y-27632 (Tocris Bioscience, Bristol, England) and with 1% FBS for 0.5h-1h at 37 °C. After washing, filtration through a 70-μM cell strainer (Biosharp, Hefei, China), and centrifugated, cell pellets were embedded in Matrigel (Corning) plated in 24-well plates and cultured in medium containing Advanced DMEM/F12 (Thermo Fisher Scientific, MA, USA), Noggin (R&D Systems,MN,USA), R-Spondin (R&D Systems), EGF (R&D Systems.), Glutamax (Invitrogen), HEPES (Invitrogen), N-2 additive (Invitrogen), B27 cell culture additive (Thermo Fisher Scientific), N-acetylcysteine (Tocris Bioscience), Nicotinamide (Tocris Bioscience), A83-01 (Tocris Bioscience) and SB202190 (Sigma-Aldrich, Darmstadt, Germany). The medium was refreshed approximately every 4 days.

### 2.10 Statistical methods

To minimize bias, group allocation was performed using a random number table. Furthermore, investigators involved in experimental procedures, data collection, and analysis were blinded to group assignments. Transcriptome data were preprocessed for further analysis. First, transcriptome data were standardized (RSEM for TCGA; Log2(MAS5) for GSE31210; RMA for GSE30219). To enable cross-dataset analysis, the standardized data were normalized by the function (x-min[x])/(max[x]-min[x]). Furthermore, normalized genes with a higher variation than the upper quartile of the mean absolute deviation (MAD) were selected and scaled by Z transformation for further analysis. The ConsensusClusterPlus package was applied for clustering analysis (30). UMAP (UMAP package) and t-distributed stochastic neighbor embedding (t-SNE) (Rtsne package) were used to evaluate the clustering results (31, 32). Kaplan–Meier curves were used to visualize survival probabilities, with the log-rank test employed for statistical comparison (the Benjamini–Hochberg method was applied for P−value adjustment in multi−group comparisons to balance false positives and false negatives). The Cox proportional hazards regression model was utilized for risk factor assessment, and Least Absolute Shrinkage and Selection Operator (LASSO) regression models were applied for variable selection given their effectiveness in handling high−dimensional complex data (using the survival and survminer packages). Only patients with survival times of more than 30 days were included in the prognostic analysis to avoid the interference of perioperative death (33). The DESeq2 package was used for differential gene analysis under the conditions of adj. p<0.05 and abs(log fold change (logFC))>1 (34). Receiver operating characteristic (ROC) curves were used for diagnostic analysis (pROC package). Gene set enrichment analysis (GSEA) was performed to assess biological significance based on Hallmark databases and KEGG databases. The ggplot2 package was used for graphing. All statistical analyses were performed using R software, with two-sided p values (or adj. p values) <0.05 considered statistically significant.

## 3 Results

### 3.1 Workflow for the molecular classification and evaluation of stage IB NSCLC

**Supplementary Figure 1** shows the study design. First, for TCGA-NSCLC, after standardization and normalization, hypervariable genes with an MAD above the upper quartile (0.1393) were selected (5006/20472). After further Z-scaling, stage IB patients were classified into two molecular types (IB1, 48; IB2, 71) by consensus clustering. UMAP and t-SNE were used to evaluate the classification performance. The prognostic significance of this molecular classification was assessed. First, we evaluated the effectiveness of the 8th edition TNM staging system in addressing the heterogeneity of stage IB NSCLC in the SEER database (survival time >1 month). The differences in DSS between stage IB (3 cm-4 cm) (7th edition stage IB, 8th edition stage IB) patients (4021), stage IB (4 cm-5 cm) (7th edition stage IB, 8th edition stage IIA) patients (2117), stage IA patients (14836), and stage II patients (10125) (stage IIA, 5036; stage IIB, 5012; not specified, 77) were compared. Next, we evaluated our staging in TCGA-NSCLC, after excluding patients with survival times of less than 30 days, 94 IB patients from the TCGA-NSCLC were evaluated to assess the differences in overall survival (OS) and disease-free survival (DFS). We also compared the prognosis between stage IB1/2 patients and stage IA or stage II (IIA and IIB) patients. After excluding patients with a survival time of less than 30 days, the analysis included 37 stage IB1 patients, 57 stage IB2 patients, 109 stage IA patients, and 103 stage II patients (comprising 62 stage IIA patients, 39 stage IIB patients, and 2 patients with an unspecified IIA/IIB subclassification). Finally, differentially expressed genes (DEGs) between stage IB1 and IB2 were filtered by DFS analysis and further included in LASSO models to assess the predictive potential for stage IB1/2 classification and prognosis evaluation, and 3 genes were selected. The GSE31210 dataset was used for validation. Fifty-three stage IB patients underwent classification according to the above procedures (IB1, 21; IB2, 32). A total of 108 stage IA patients, 21 stage IB1 patients, 32 stage IB2 patients, and 39 stage II patients with complete prognostic information were used for survival assessment. Our dataset (30 stage IB patients), GSE30219 (13 stage IB patients), pathological tissues and PDOs of stage IB NSCLC, and cell lines were used to investigate clinical relevance and biological functions of critical genes involved in the malignant progression of stage IB NSCLC.

### 3.2 Molecular typing of stage IB NSCLC

**Figure 1:**
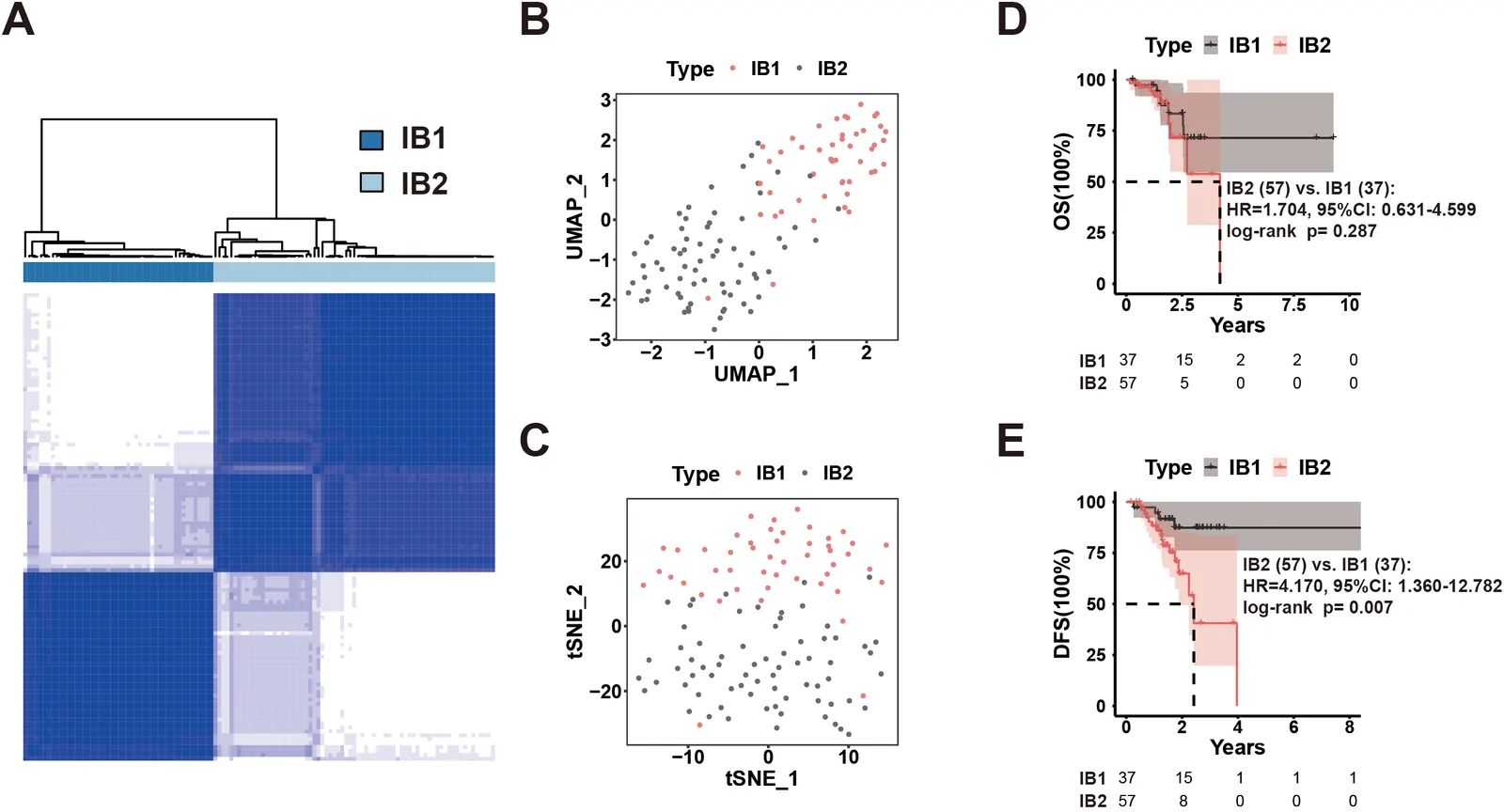
Molecular typing of stage IB NSCLC patients. **(A)** Consistent clustering of stage IB TCGA-NSCLC patients into two types; **(B)** UMAP and **(C)** t-SNE demonstrations of the classification efficacy of IB1/2 patients; **(D)** OS and **(E)** DFS assessments between stage IB1 and stage IB2.

After data preprocessing, we conducted consensus clustering for 119 TCGA-NSCLC stage IB patients according to the similarity of gene expression profiles and divided the patients into two categories: stage IB1 and stage IB2 (**Figure 1A**). UMAP and t-SNE can visually display high-dimensional data by projecting data points with similar distances onto a two-dimensional plane. In this study, UMAP and t-SNE dimensionality reduction showed distinct classification of stage IB1/2 (**Figures 1B, C**). We further compared prognostic differences between stage IB1 and stage IB2 patients. The median OS time was 51 months in stage IB2, and not reached in stage IB1, but the difference in OS did not reach statistical significance (HR, 1.704; 95% CI, 0.631 to 4.599) (**Figure 1D**). There was a significant DFS advantage in stage IB1 type. The median DFS time was 29.4 months in stage IB2 and not reached in stage IB1 (HR, 4.170; 95% CI, 1.360 to 12.782) (**Figure 1E**). These results suggest that stage IB NSCLC has strong heterogeneity, and further classification of stage IB is needed, especially to guide precise treatment.

### 3.3 Clinical significance of the change in stage IB NSCLC from the AJCC 7th edition system to the 8th edition system

For stage IB NSCLC, stage IB of the 7th edition was defined as T >3 cm and ≤ 5 cm, N0 and M0, while stage IB of the 8th edition was defined as T >3 cm and ≤ 4 cm, N0, and M0. The most significant change was that patients with T> 4 cm and ≤ 5 cm, N0, and M0 were reclassified as stage IIA. Does this classification completely solve the problem of the heterogeneity of stage IB NSCLC? Is it possible to distinguish between patients who need surgery alone and those who need aggressive adjuvant postoperative care? We explored over 30,000 NSCLC patients in the SEER database. We found that the prognosis of stage IB (4 cm-5 cm) patients (2117) was indeed significantly worse than that of stage IB (3 cm-4 cm) patients (4021) (HR: 1.275, 95% CI: 1.170 to 1.390, log-rank p <0.001) (**Supplementary Figure 2A**). However, it should be noted that the prognosis of stage IB (3 cm-4 cm) patients (4021) was still significantly worse than that of stage IA patients (14836) (HR: 1.851, 95% CI: 1.736 to 1.974, log-rank p <0.001) (**Supplementary Figure 2A**). It is suggested that stage IB patients (3 cm-4 cm) may still not be able to be adequately managed with the same treatment as stage IA patients (surgical treatment alone). Meanwhile, stage IB (4 cm-5 cm) patients (2117) had a better prognosis than stage II patients (10125) (HR: 0.794, 95% CI: 0.737 to 0.854, log-rank p <0.001) (**Supplementary Figure 2B**). Subgroup analysis also showed that patients with stage IB (4 cm-5 cm) had significantly better outcomes than patients with stage IIA (5036) and stage IIB (5012) (HR: 0.869, 95% CI: 0.803 to 0.941, log-rank p <0.001; HR: 0.734, 95% CI: 0.678 to 0.793, log-rank p <0.001) (**Supplementary Figures 2C, D**). Thus, patients with stage IB (4 cm-5 cm) may not be adequately managed with stage II (IIA and IIB) treatment regimens (postoperative adjuvant therapy). These results suggest that the issue of heterogeneity in stage IB NSCLC has not been completely resolved by the eighth edition of the staging system.

### 3.4 Clinical significance of the molecular classification of stage IB NSCLC

**Figure 2:**
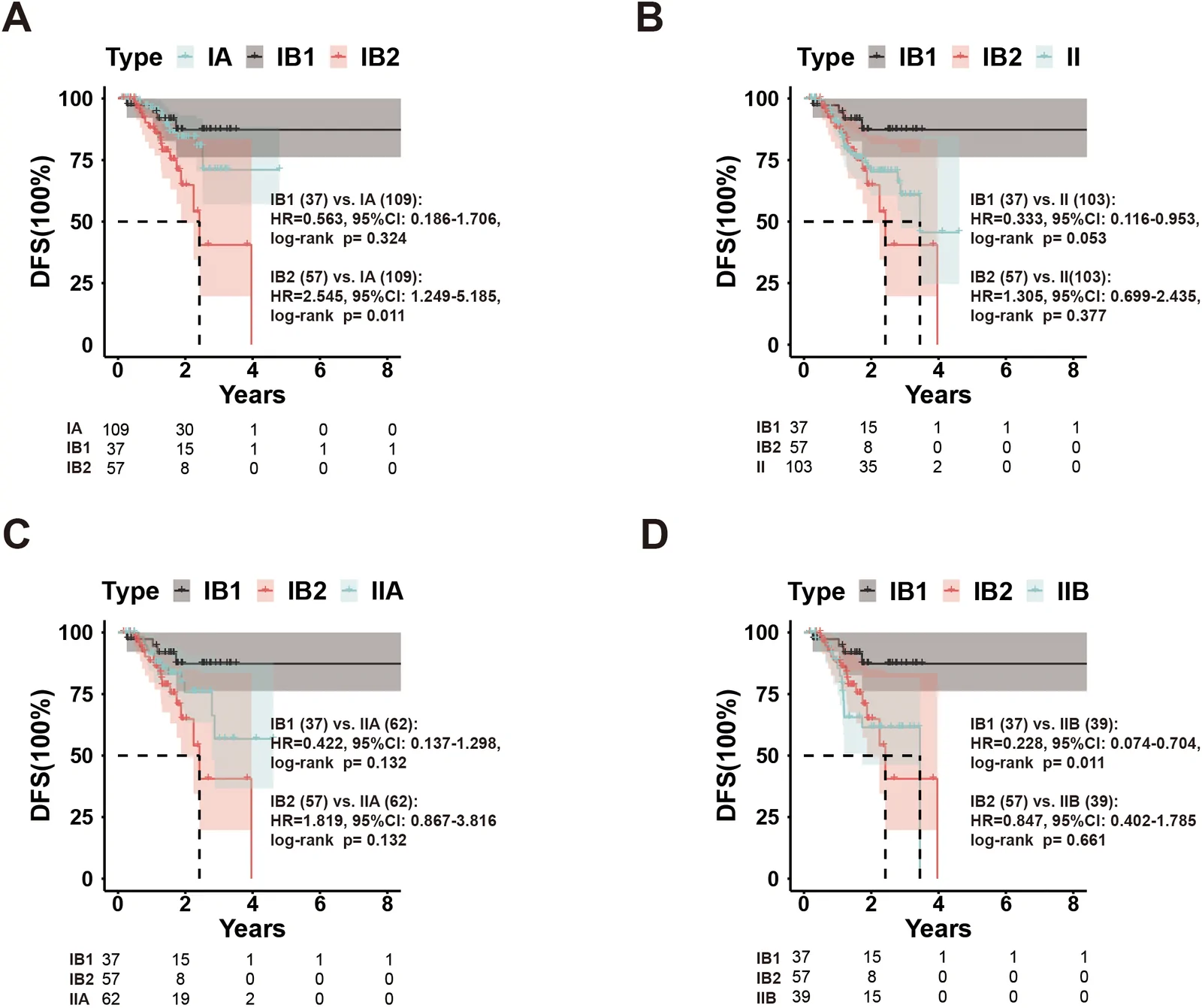
Evaluation of the clinical significance of molecular typing in patients with stage IB NSCLC. DFS assessment between stage IB1/2 patients and stage IA patients **(A)**, stage II patients **(B)**, stage IIA patients **(C)**, and stage IIB **(D)** patients.

Next, we compared prognostic differences between our molecular classification of stage IB (IB1/2) and stages IA, IIA, and IIB to evaluate the clinical characteristics of stage IB for choosing possible precise clinical treatment. We found that stage IB1 patients showed DFS comparable to that of stage IA patients (HR = 0.563, 95% CI: 0.186-1.706, log-rank p=0.324), suggesting that stage IB1 patients may have similar prognostic characteristics to stage IA patients, calling for similar clinical procedures, that is, without the need for postoperative adjuvant chemotherapy (**Figure 2A**). However, stage IB2 patients had a much worse DFS than stage IA patients (HR = 2.545, 95% CI: 1.249-5.185, log-rank p=0.011), suggesting that stage IB2 patients may need aggressive interventions such as postoperative adjuvant chemotherapy, not just surgical excision (**Figure 2A**). In addition, stage IB2 patients had a similar DFS to stage II patients (p=0.377), and the median DFS of stage IB2 patients was shorter than that of stage II patients (29.4 months vs. 41.9 months), suggesting that the clinical treatment of stage IB2 patients can be similar to that of stage II patients; that is, adjuvant therapy should be carried out in a timely manner after surgery (**Figure 2B**). Stage IB1 tended to have a longer DFS than stage II, although it presented no statistically significant advantage in log-rank test (p=0.053), stage IB1 exhibited lower risk in prognosis compared to stage II (HR, 0.333; 95% CI, 0.116 to 0.953) (**Figure 2B**). Increased sample size and prolonged follow-up may further highlight the gap between stage IB1 and stage II, suggesting that the two should be treated differently. Considering that high heterogeneity in stage II patients might mask the actual statistical significance, we subdivided stage II into stage IIA and stage IIB and compared stage IB1/2 with stages IIA and IIB. We did not find a statistically significant difference between stage IIA and stage IB1/2 (p=0.132), but stage IB2 had a trend toward inferior DFS compared with stage IIA, and stage IB1 had a tendency of much longer DFS than stage IIA (**Figure 2C**). These tendencies exhibited statistical significance in stage IIB. Stage IB2 patients had a similar DFS to stage IIB patients (HR = 0.847, 95% CI: 0.402-1.785, log-rank p=0.661), but stage IB1 patients had a much longer DFS than stage IIB patients (HR = 0.228, 95% CI: 0.074-0.704, log-rank p=0.011) (**Figure 2D**). These results once again suggest that stage IB2 has a prognosis similar to that of stage IIB and may require aggressive postoperative adjuvant therapy, while stage IB1 has a better prognosis than stage IIB and may not require aggressive postoperative treatment.

### 3.5 Validation of molecular classification of stage IB NSCLC

**Figure 3:**
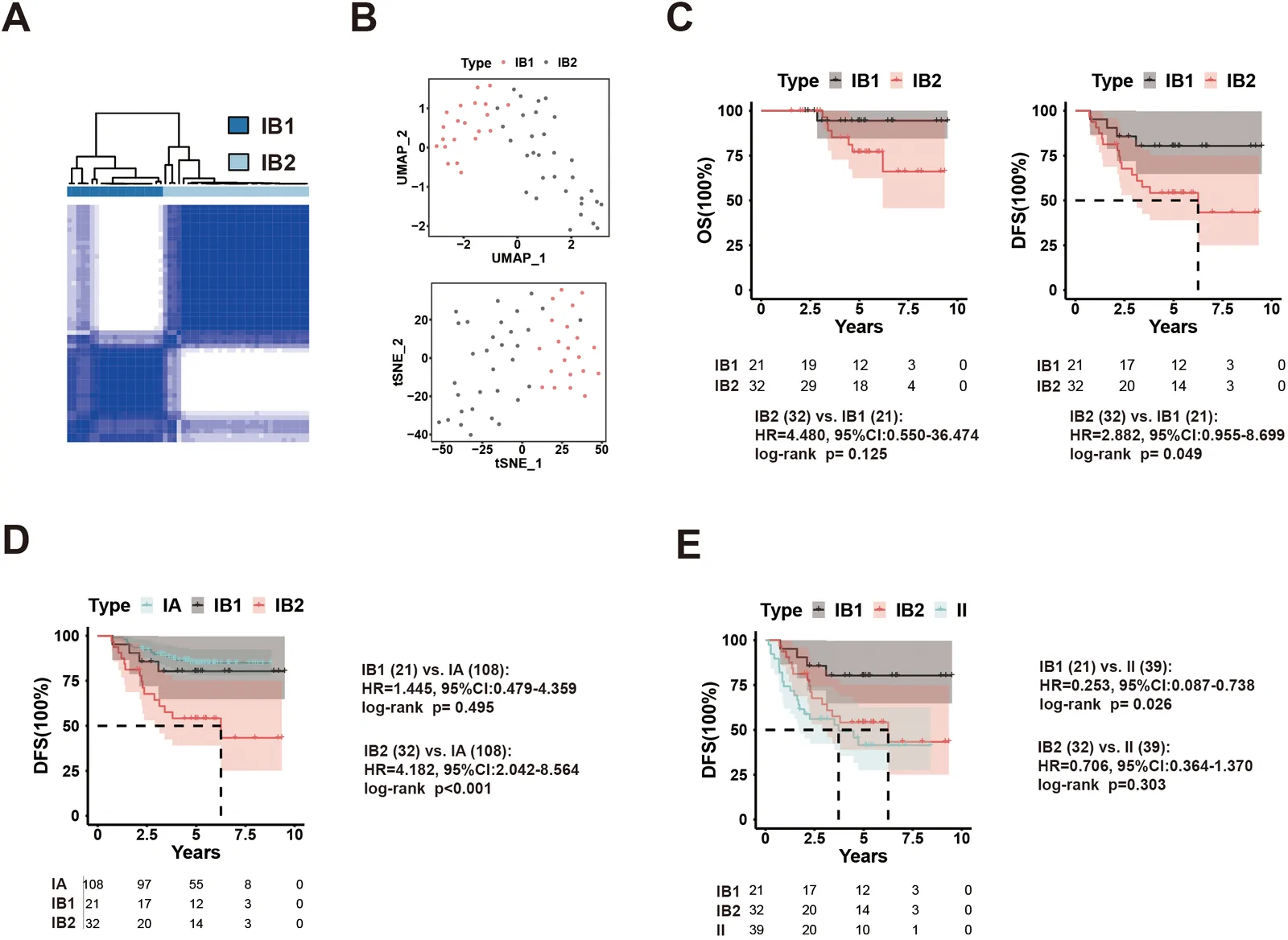
Validation of molecular typing of stage IB NSCLC in GSE31210 dataset. **(A)** Consensus clustering of stage IB NSCLC in GSE30210 into two types (IB1/2); **(B)** usage of UMAP (upper) and t-SNE (low) to exhibit classification efficacy of stage IB1/2; **(C)** OS (left) and DFS (right) evaluation between stage IB1 and IB2; DFS evaluation between stage IB1/2 patients and stage IA **(D)** and II **(E)** patients.

To assess the universality of our molecular typing, we used GSE31210 for validation. Using the above consensus clustering method, we found that stage IB patients in GSE31210 had a distinct molecular classification with clinical significance in stage IB (**Figures 3A–C**). These stage IB types in GSE31210 also showed potential clinical implications; that is, the stage IB type patients with a more favorable prognosis had a similar DFS compared with stage IA patients, and the worse-prognosis stage IB type patients had a similar DFS compared with stage II patients (**Figures 3D, E**).

### 3.6 Gene signatures for the molecular typing of stage IB NSCLC

**Figure 4:**
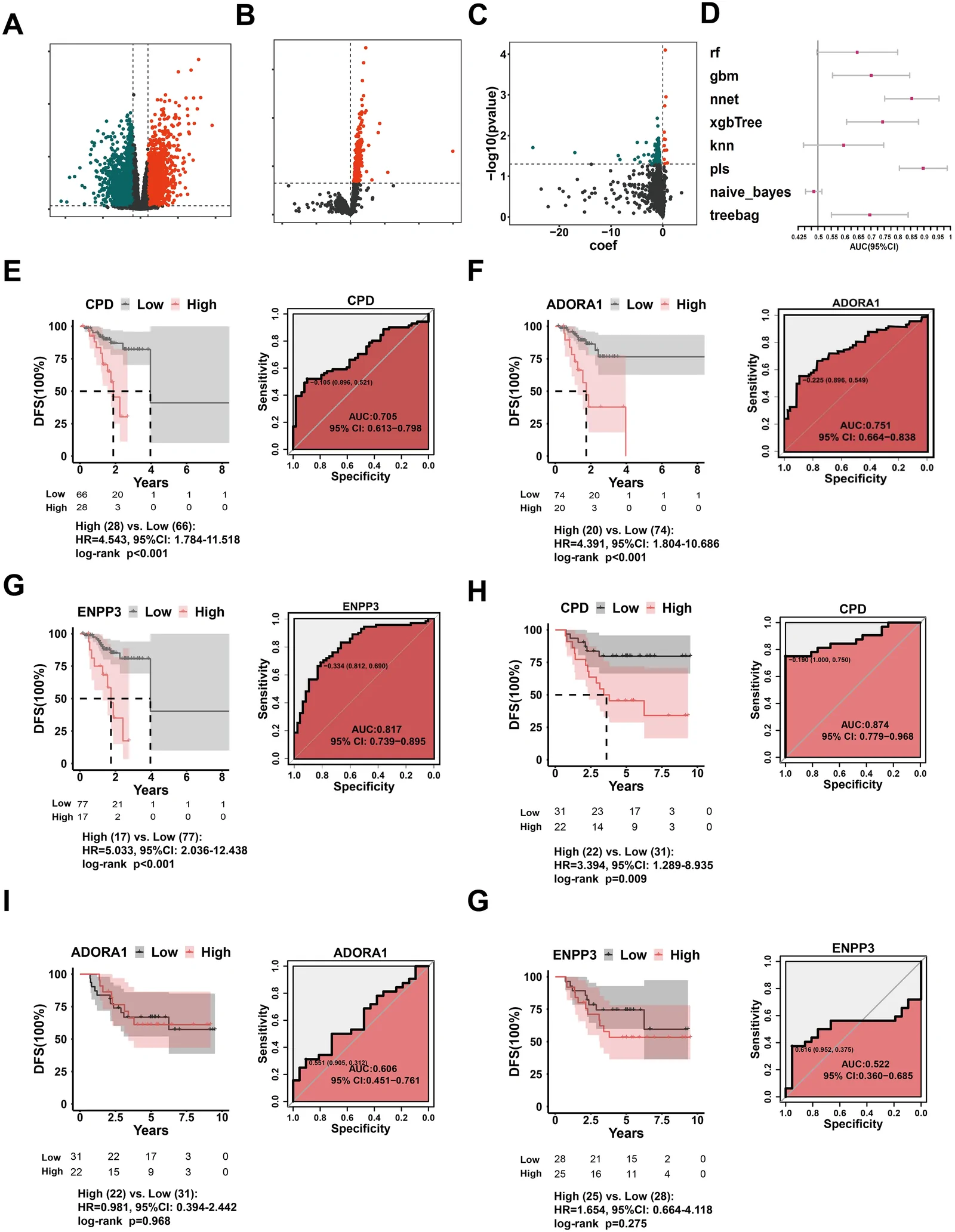
Biomarkers for the molecular typing of stage IB NSCLC. **(A)** Differential gene analysis of stage IB1 and IB2 patients; **(B)** Screening out hazardous prognostic factors from upregulated genes **(C)** and protective factors from downregulated genes; **(D)** Predictive efficacies of eight machine learning algorithms to distinguish IB1 and IB2; CPD **(E)**, ADORA1 **(F)** and ENPP3 **(G)** for distinguishing stage IB molecular subtypes (right) and prognosis prediction of stage IB NSCLC (left) in training set; Efficacy of CPD **(H)**, ADORA1 **(I)** and ENPP3 **(G)** for molecular diagnostic assessment (right) and prognostic prediction (left) of stage IB NSCLC in validation set.

We sought to identify a gene signature for stage IB molecular classification, especially for survival prediction. First, we found 4355 DEGs between stage IB2 and stage IB1, with 2423 upregulated DEGs and 1932 downregulated DEGs in the poor-prognosis stage IB2 type in TCGA cohort (**Figure 4A**). Furthermore, we evaluated the ability of these DEGs to predict DFS, and we obtained 300 upregulated DEGs with hazardous prediction and 114 downregulated DEGs with protective efficacy (**Figures 4B, C**).

Next, we used eight machine learning algorithms based on these DEGs with prognostic significance to distinguish IB1 and IB2, while TCGA-NSCLC was used as a training set, and GSE31210 was used as a validation set (random forest, rf; stochastic gradient boosting, gbm; neural network, nnet; extreme gradient boosting, xgbtree; K-nearest neighbor, knn; bagged classification and regression tree, treebag; partial least squares, pls and naive bayes, naive_bayes) (**Figure 4D**). To ensure robustness and address potential overfitting, we further conducted an internal 4-fold cross-validation. With this more rigorous K-fold cross-validation, the performance of all models significantly improved and stabilized, demonstrating the strong inherent predictive power of the prognostic DEGs (**Supplementary Figures 3A–D**).

Furthermore, we used a LASSO regression model for dimensionality reduction to select the most important genes for DFS prediction (**Supplementary Figure 3A**). Based on lambda.min and coef, three genes (adenosine A1 receptor, ADORA1; carboxypeptidase D, CPD; and ectonucleotide pyrophosphatase/phosphodiesterase 3, ENPP3) were finally selected, which all showed fine molecular classification and survival prediction abilities in the training set (**Figures 3E–G**). Finally, we used the gene signature (CPD, ADORA1, and ENPP3) to evaluate stage IB patients in the validation set. We found that only CPD had fine molecular classification abilities (AUC: 0.874), and high CPD expression could represent the poor-prognosis stage IB type and predict shorter DFS (**Figures 4H–G**; **Supplementary Figures 4A–L**). Overall, we believe CPD may function as a good molecular biomarker for distinguishing stage IB NSCLC types for precise clinical procedures.

### 3.7 Risk stratification of CPD in stage IB NSCLC

**Figure 5:**
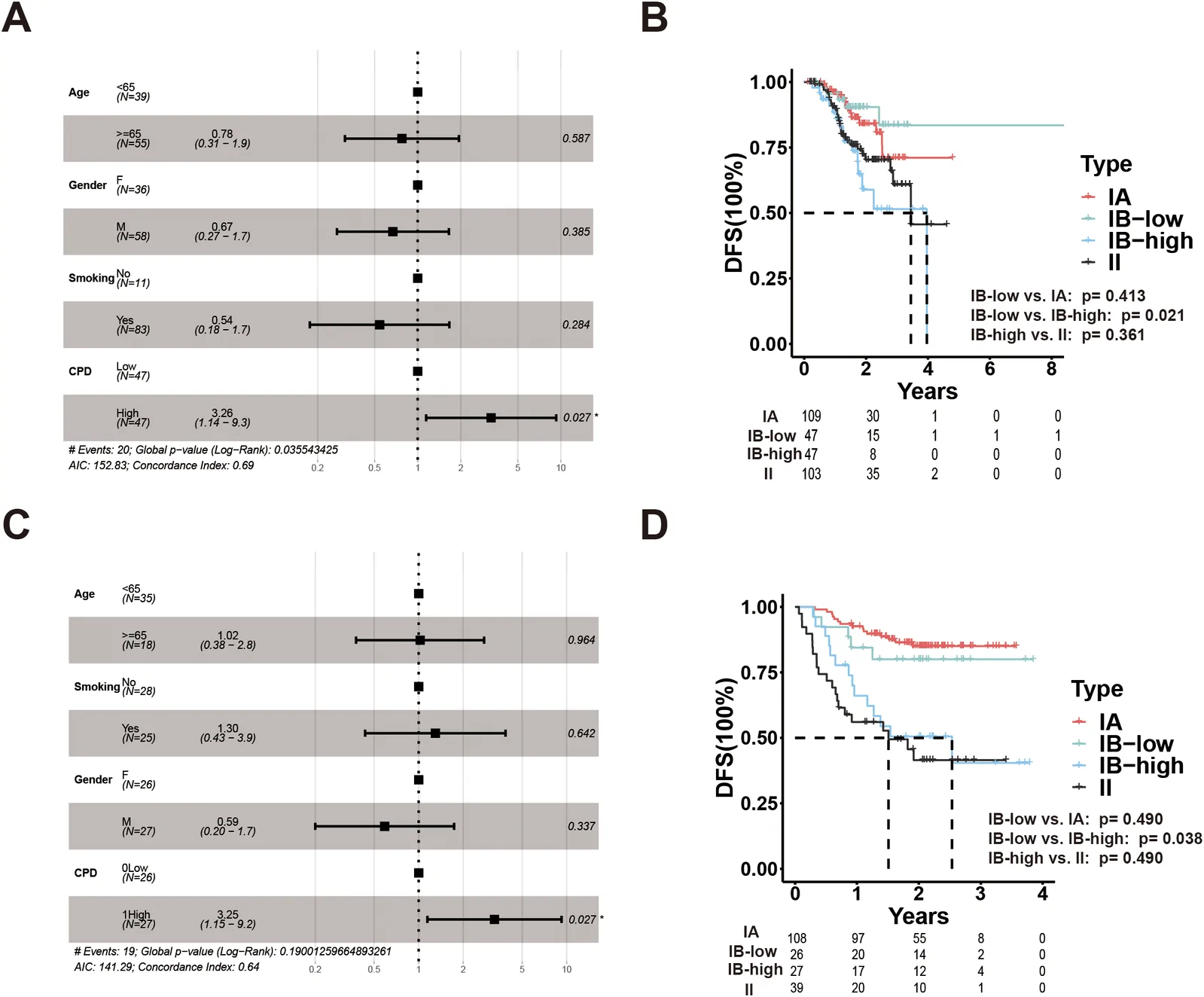
Risk stratification of CPD in stage IB NSCLC. Independent risk prediction of CPD expression in DFS in two stage IB NSCLC datasets [**(A)**, TCGA-NSCLC; **(C)**, GSE30210]; DFS curves among two risk types of stage IB NSCLC patients divided by median of CPD expression (IB-low and IB-high), stage IA and stage II patients in two stage IB NSCLC datasets [**(B)**, TCGA-NSCLC; **(D)**, GSE30210].

Furthermore, we demonstrated that high CPD expression in tumor tissues was an independent predictor of risk for stage IB DFS both in TCGA and GSE31210 datasets (**Figures 5A, C**). We also found that CPD had a more thorough stratification of stage IB risk both in TCGA and GSE31210 datasets (**Figures 5B, D**). That is, when stage IB patients were stratified by CPD content, the prognosis of stage IB patients with low CPD expression had a similar DFS to stage IA patients, for whom postoperative adjuvant chemotherapy is unnecessary, while the prognosis of stage IB patients with high CPD expression had a similar DFS to stage II patients, for whom adjuvant therapy should be administered immediately after surgery. The results in both datasets are consistent and highly significant, regardless of whether using the data-based optimal threshold method or the continuous variable analysis method (**Supplementary Figures 5A–F**).

### 3.8 CPD is highly expressed in stage IB NSCLC and is positively associated with malignant prognosis

**Figure 6:**
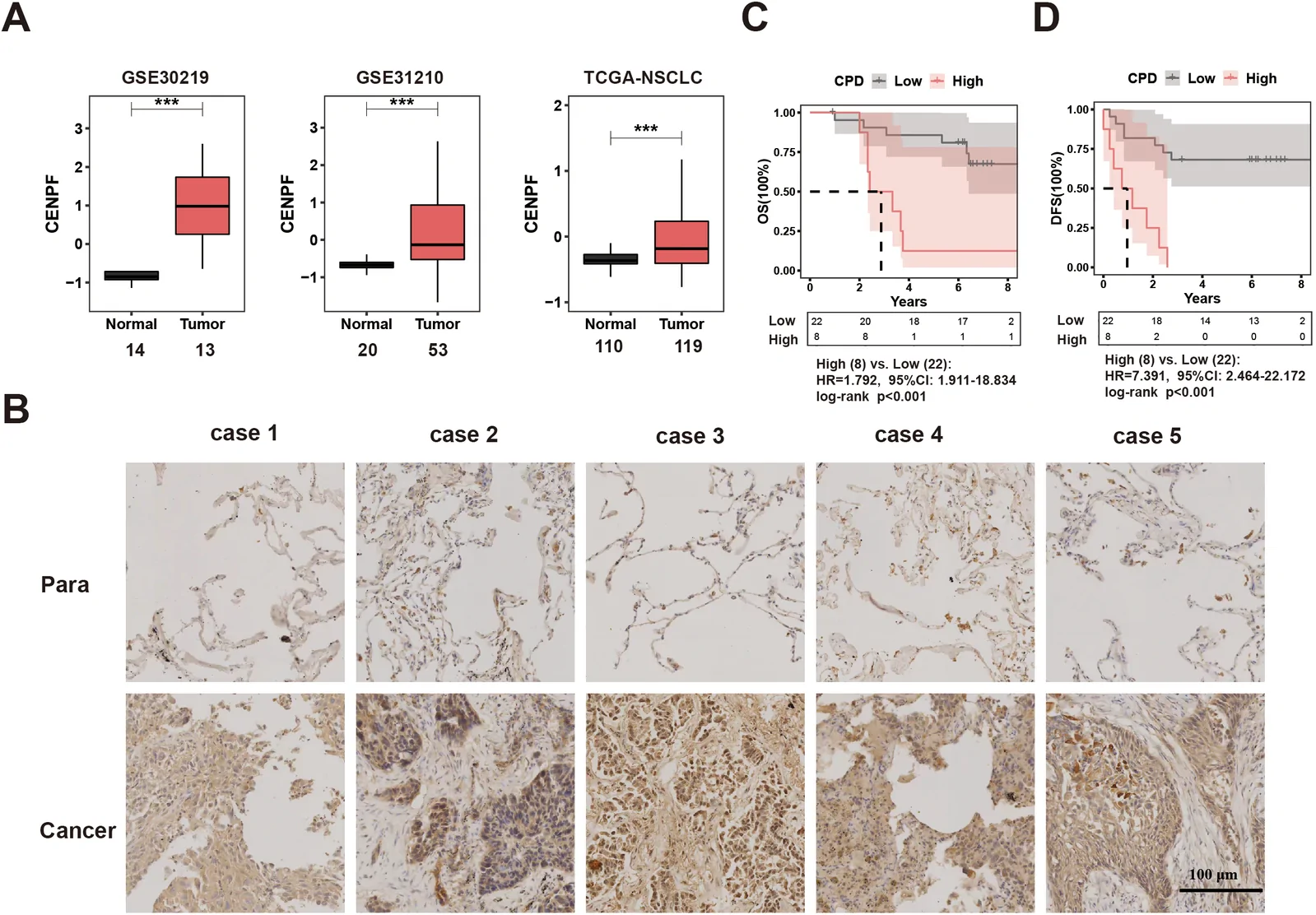
CPD possesses high expression profile in stage IB NSCLC and predicts poorer prognosis. **(A)** The expression of CPD in cancer and normal tissues from stage IB NSCLC of three transcriptional datasets; **(B)** Pathological illustrations of CPD protein expression in cancer and para-cancer tissues from stage IB NSCLC; CPD for OS **(C)** and DFS **(D)** prediction of stage IB NSCLC. "***" statistical significance with p<0.001.

Given the impact of CPD on the high- and low- risk classification of stage IB NSCLC, we further explored its role in this disease. We first explored the expression of CPD in stage IB NSCLC. In three stage IB NSCLC public datasets (GSE30219, GSE31210, TCGA-NSCLC), we found that the transcriptional level of CPD in cancer tissues was significantly higher than that in normal tissues (**Figure 6A**). Meanwhile, we collected stage IB NSCLC specimens in our department, and demonstrated that CPD protein levels in cancer cells were higher than those in tumor-adjacent tissues by immunohistochemistry (**Figure 6B**). Through qPCR, we quantified the CPD gene expression levels in stage IB NSCLC dataset of our department, and also confirmed high CPD expression was associated with a poorer survival rate (**Figures 6C, D**). Therefore, the above results suggest that CPD possesses high transcriptional and protein expression in stage IB NSCLC, and high expression of CPD indicates a malignant prognosis.

### 3.9 CPD promotes malignant progression of stage IB NSCLC

**Figure 7:**
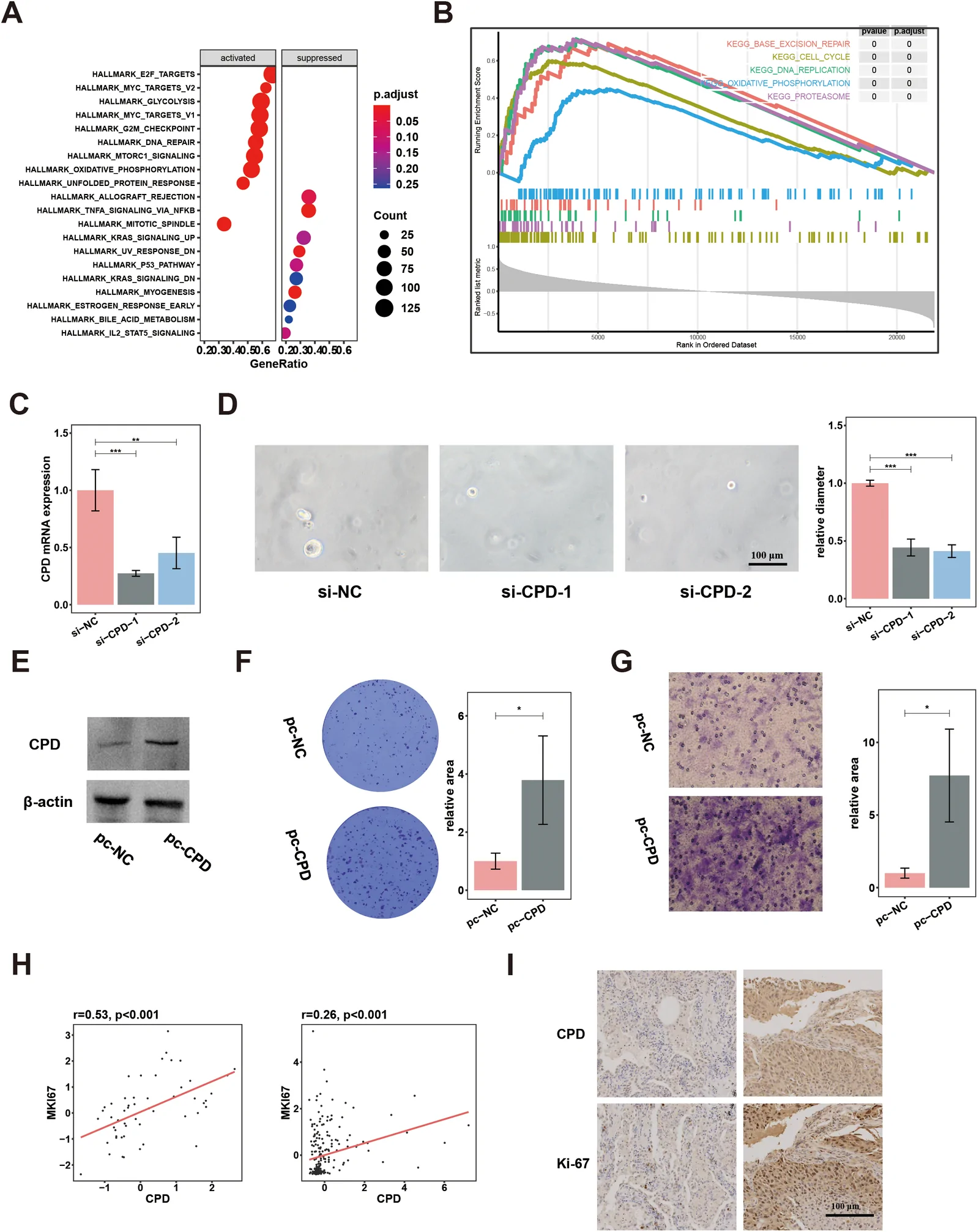
Biological function of CPD in malignant progression of stage IB. Bio-enrichment of CPD expression by GSEA of CPD associated genes (ranked in correlation index order) in Hallmark database **(A)** and KEGG database **(B)**; **(C)** Establishment of knockdown model of CPD in PDO of stage IB NSCLC and qPCR was used to examined CPD expression; **(D)** Growth inhibition of CPD in PDO of stage IB NSCLC; typical diagram (left) and statistical significance (right); **(E)** Establishment of overexpression model of CPD in H1975 cell lines and WB was used to examined CPD expression; **(F)** The role of CPD in plate colony in H1975 cell lines, typical diagram (left) and statistical significance (right); **(G)** The role of CPD in cell migration ability in H1975 cell lines, typical diagram (left) and statistical significance (right); **(H)** Correlation between CPD expression and MKI67 expression in stage IB NSCLC datasets (left, GSE31210; right, TCGA-NSCLC); **(I)** Typical pathological illustrations of the correlation between CPD protein expression and Ki-67 protein expression in specimens of stage IB NSCLC. "*" indicates p < 0.05, "**" indicates p < 0.01 and "***" indicates p < 0.001.

We then explored the biological role of CPD in stage IB NSCLC. By GSEA of CPD-associated genes (ranked in correlation coefficient) in stage IB NSCLC, we found that CPD expression in stage IB NSCLC tissues was positively correlated with malignant phenotypes and signaling pathways such as cell proliferation and E2F signaling, in both the Hallmark database and KEGG database (**Figures 7A, B**). PDO preserves the histological and genetic characteristics of tumors, making them an ideal model for tumor research. We established a PDO model of stage IB NSCLC and constructed a CPD knockdown model through RNA interference (**Figure 7C**). We found that CPD knockdown significantly inhibited the growth of stage IB NSCLC PDOs (**Figure 7D**; **Supplementary Figure 6**). We further constructed a CPD overexpression model in the NSCLC cell line H1975 (**Figure 7E**). Through colony formation assays, we found that CPD overexpression significantly promoted cell cloning ability (**Figure 7F**). Transwell assays also proved that CPD overexpression significantly promoted cell migration ability (**Figure 7G**), and we further confirmed this finding in H358 cells (**Supplementary Figures 7A, B**). In two stage IB NSCLC datasets (TCGA-LUAD, GSE31210), we confirmed a significant positive correlation between CPD and MKI67 expression levels (a classic malignant proliferation marker) (**Figure 7H**). In pathological samples of stage IB NSCLC, we also demonstrated a positive correlation between CPD and Ki-67 protein expression by immunohistochemistry (**Figure 7I**). These results indicate that CPD significantly promotes the malignant biological features of stage IB NSCLC.

## 4 Discussion

The heterogeneity of stage IB NSCLC and its treatment have remained the focus of academic attention. The JLCRG study indicated that postoperative adjuvant chemotherapy can significantly improve the prognosis of stage IB LUAD, but in this study, chemotherapy (oral uracil/tegafur) was not commonly used (12). Several large clinical trials and meta-analyses (IALT, ANITA trial, JBR.10 trial and LACE) all indicated the benefits of common adjuvant chemotherapy (platinum-containing doublet) for early-stage NSCLC patients, but stage IB disease failed to exhibit significance in the subgroup analysis (13–16). Nevertheless, the CALGB 9633 trial and long-term analysis of the JBR.10 trial demonstrated that stage IB patients with tumors ≥ 4 cm could benefit from adjuvant chemotherapy (3, 4). Subsequent studies also suggested that stage IB patients with pleural invasion or solid/micropapillary components have a poorer prognosis (1, 2).

The results of the above clinical trials elevated some patients with stage IB NSCLC in the 7th edition staging system (T > 4 cm and ≤ 5 cm, N0, M0) to stage IIA in the 8th edition. However, in the 8th edition staging system, the heterogeneity of stage IB has not been fully resolved. As stage IB remains intermediate between stage II and stage IA, the appropriate treatment approach continues to be unclear (8). Besides, the existing high-risk factors based largely on macroscopic pathological features fail to effectively classify high-risk types of stage IB NSCLC, for even if they exhibit the same morphological and pathological characteristics, their internal biological characteristics and molecular mechanisms still have great heterogeneity. Therefore, we attempted to perform molecular typing of stage IB NSCLC in this study. We still decided to classify stage IB patients based on the 7th edition staging system (broader coverage including stage IB in the 8th edition) to better aid clinical decision-making.

We divided stage IB NSCLC into stage IB1 and IB2 based on the differences in gene expression profiles. We found that stage IB2 patients had a worse prognosis than stage IB1 patients. Stage IB2 patients had a similar prognosis to stage II patients but a worse prognosis than stage IA patients, suggesting that stage IB2 is more aggressive than stage IA and that the treatment of these patients should be similar to that of stage II patients; that is, active adjuvant treatment is warranted. However, the prognosis of stage IB1 patients was similar to that of stage IA patients, suggesting that these patients can be observed postoperatively. Although there was no significant difference between stage IB1 patients and stage II patients in our study, we cannot easily conclude that stage IB1 patients and stage II patients should be treated similarly. On the one hand, the DFS of stage IB1 patients was longer than that of stage II patients, and statistical significance of this advantage may appear with prolonged follow-up. On the other hand, there is large heterogeneity in stage II patients. In this study, stage II patients included 62 stage IIA patients and 39 stage IIB patients, and 2 patients were not specified. The proportion of stage IIA patients was relatively large, and the median DFS of stage IB1 and stage IIA patients were not reached, which may mask the survival advantage of stage IB1 patients over stage IIA patients or overall stage II patients. Prolonging the follow-up time is likely to make the advantage trend statistically significant. The subgroup analysis showed that the prognosis of stage IIB patients was significantly worse than that of stage IB1 patients but similar to that of stage IB2 patients.

It is noteworthy that the IB2 subtype demonstrated a statistically significantly worse DFS compared to IB1, while the difference in OS showed a consistent trend but did not reach statistical significance. This discrepancy is frequently observed in oncology and can be attributed to several factors. OS can be confounded by effective subsequent therapies administered after disease recurrence, which may prolong survival in the high-risk IB2 group and thereby attenuate the initial prognostic difference. Furthermore, the clinical significance of a robust DFS distinction should not be understated, especially in early-stage disease. Identifying a patient subgroup with a high risk of recurrence is paramount for optimizing post-operative management strategies, such as consideration of adjuvant therapy, with the ultimate aim of preventing recurrence and improving cure rates.

Although the machine learning models demonstrated excellent classification performance under robust validation, we strategically focused on the minimal CPD signature for its superior clinical utility and interpretability. This parsimony is advantageous for developing a diagnostic assay and for facilitating functional validation of its role in the IB2 subtype. CPD, traditionally considered a proteinase that cleaves C-terminal arginine and lysine residues from its substrates, has recently been shown to exhibit oncogenic potential in cancers (17). The classification of stage IB heterogeneity by molecular typing, the clinical prediction effect of high- and low-risk IB subtypes after molecular typing, and the use of CPD as a molecular typing marker for stage IB NSCLC were all verified in the GSE31210 dataset, confirming the generalizability of the above classification.

In view of the clinical significance of CPD in risk stratification of stage IB NSCLC, we further analyzed the expression and biological role of the CPD gene in stage IB NSCLC. CPD can hydrolyze the C-terminal amino acids of polypeptides, perform post-translational modifications on proteins, and then regulate protein activity, affecting biological processes such as microtubule assembly, signal transduction, and cell secretion (18–20). Recent studies have shown that CPD plays a role in promoting cancer in liver cancer, breast cancer, prostate cancer and other tumors (21–24). High expression of CPD is associated with poor prognosis, and CPD promotes tumor cell proliferation and migration (21–24).We confirmed in three transcriptional datasets that CPD is significantly over transcribed in stage IB NSCLC, and in the pathological specimens of stage IB NSCLC, the protein expression of CPD in cancerous tissues was significantly higher than that in tumor-adjacent tissues. Meanwhile, in our dataset, high expression of CPD consistently predicts a poor survival prognosis for stage IB NSCLC. Furthermore, in stage IB NSCLC, we found that CPD gene was correlated with many tumor malignant phenotypes and carcinogenic signaling networks, and it showed a typical positive relationship with malignant marker Ki-67. These results preliminarily suggest a possible cancer-promoting role of CPD gene in stage IB NSCLC. PDOs are pivotal models for cancer research, as they mimic the histological and stereochemical structural features of the original tumor (25). We established stage IB NSCLC PDOs and generated a knockdown model of CPD by RNA interference technology. We found that CPD knockdown significantly inhibited the growth stage IB NSCLC PDOs. Meanwhile, we established a model of CPD overexpression in NSCLC cell lines, and confirmed that CPD overexpression promoted the cloning and migration ability of these cells. These results suggest that CPD gene promotes the malignant progression of stage IB NSCLC, partly explaining the underlying biological mechanisms of CPD gene as a risk stratification marker for stage IB NSCLC.

Although our study is limited by its retrospective nature, the reliance on a single PDO model, and the need for further identification of direct molecular substrates, it establishes a robust foundation for CPD as a prognostic biomarker. Building on these findings, translating the proposed molecular subtypes and CPD-based stratification into routine clinical practice will require a concerted effort through sequential developmental phases. First, the prognostic and predictive value of the CPD signature necessitates prospective validation in multi-center cohorts across diverse populations and clinical settings. Concurrently, assay optimization using clinically feasible platforms (e.g., IHC or qPCR on FFPE tissues) is essential to ensure robustness, reproducibility, and seamless integration into standard pathology workflows. Subsequent steps would involve combining CPD with established clinical and molecular biomarkers to refine risk assessment, followed by health-economic evaluations to demonstrate cost-effectiveness. Ultimately, the generation of high-level evidence through prospective interventional trials will be crucial to formally evaluate whether CPD-guided adjuvant therapy decisions improve patient outcomes. By systematically addressing these translational challenges, we aim to evolve the CPD signature from a research discovery into a practical tool that enables personalized postoperative management for patients with stage IB NSCLC.

## Data Availability

Publicly available datasets were analyzed in this study. This data can be found here: https://www.ncbi.nlm.nih.gov/geo/, https://portal.gdc.cancer.gov/ and https://seer.cancer.gov/.
