# Correction: Molecular typing of stage IB non-small-cell lung cancer for precision medicine

**DOI:** 10.3389/fonc.2026.1939966

**Published:** 2026-08-11

**Authors:** Xiaoyan Li, Zitong Wan, Miaomiao Wen, Jianfei Zhu, Kaiqi Wei, Xiaohong Ji, Jiao Zhang, Guoyin Li, Jie Lei, Yangbo Feng, Yanlu Xiong, Yinxi Zhou, Shaowei Xin

**Affiliations:** 1 Department of Blood Transfusion, Shanxi Provincial People’s Hospital, Taiyuan, China; 2 Department of Thoracic Surgery, Tangdu Hospital, Fourth Military Medical University, Xi’an, China; 3 Department of Thoracic Surgery, Shaanxi Provincial People’s Hospital, Xi’an, China; 4 College of Life Science and Agronomy, Zhoukou Normal University, Zhoukou, China; 5 Academy of Medical Science, Zhengzhou University, Zhengzhou, China; 6 Innovation Center for Advanced Medicine, Tangdu Hospital, Fourth Military Medical University, Xi’an, China; 7 Department of Thoracic Surgery, The First Medical Center, Chinese People’s Liberation Army (PLA) General Hospital, Beijing, China; 8 Department of Thoracic Surgery, Air Force Medical Center, People’s Liberation Army (PLA), Beijing, China; 9 Department of Thoracic Surgery, 962 Hospital of the Joint Logistics Support Force, Harbin, Heilongjiang, China

**Keywords:** carboxypeptidase D, molecular typing, non-small-cell lung cancer, prognosis, stage IB

There was a mistake in **Figure 4** as published. In **Figure 4D**, an earlier draft version of the panel was inadvertently used in the final publication. In **Figures 4F, G**, incorrect images (ROC curves) were mistakenly placed in these panels.

**Figure 4:**
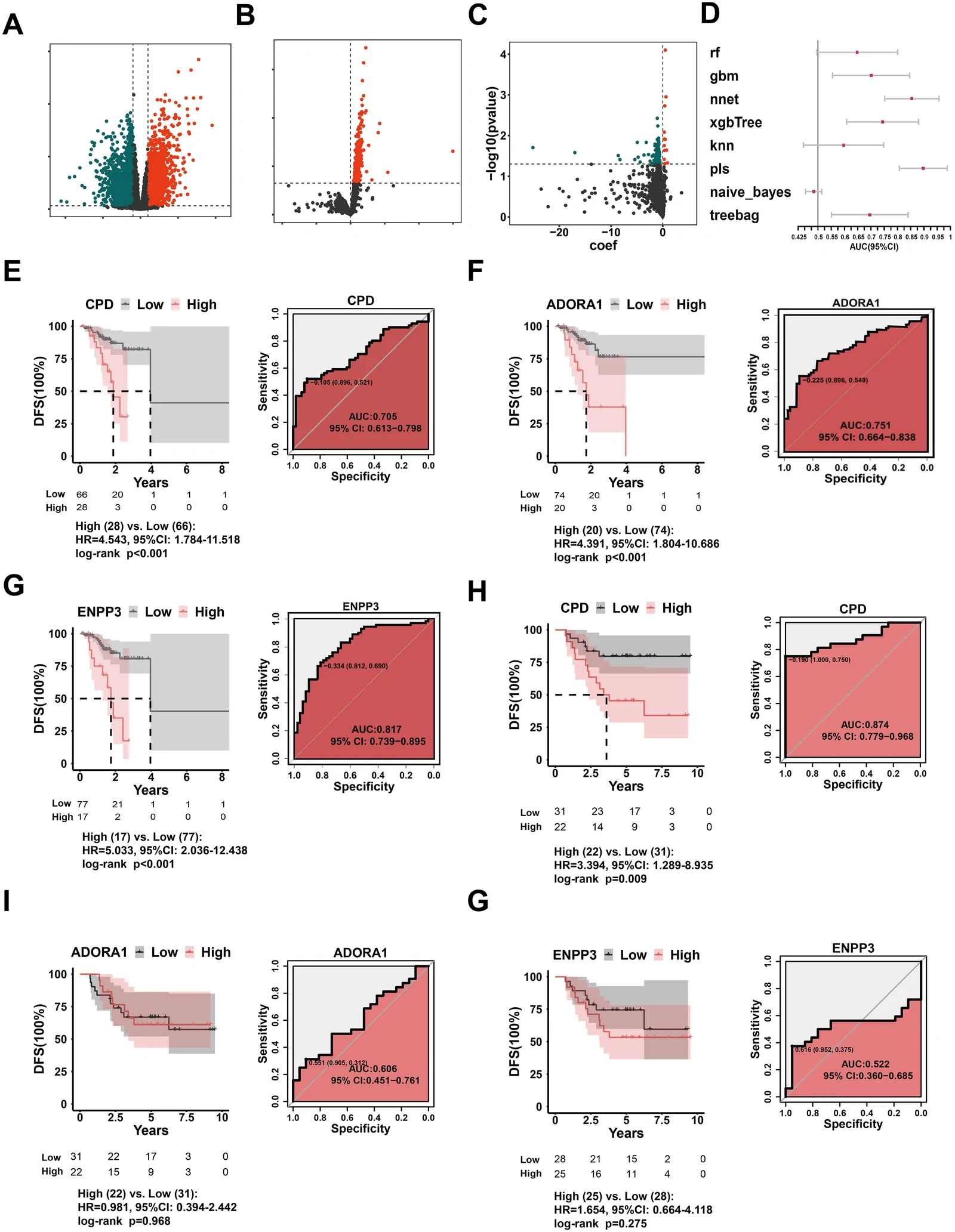
Biomarkers for the molecular typing of stage IB NSCLC. **(A)** Differential gene analysis of stage IB1 and IB2 patients; **(B)** Screening out hazardous prognostic factors from upregulated genes **(C)** and protective factors from downregulated genes; **(D)** Predictive efficacies of eight machine learning algorithms to distinguish IB1 and IB2; CPD **(E)**, ADORA1 **(F)** and ENPP3 **(G)** for distinguishing stage IB molecular subtypes (right) and prognosis prediction of stage IB NSCLC (left) in training set; Efficacy of CPD **(H)**, ADORA1 **(I)** and ENPP3 **(G)** for molecular diagnostic assessment (right) and prognostic prediction (left) of stage IB NSCLC in validation set.

The corrected **Figure 4** appears below.

Affiliation Department of Thoracic Surgery, 962 Hospital of the Joint Logistics Support Force, Harbin, Heilongjiang, China was incorrectly listed as Department of Pathology, 962 Hospital of the Joint Logistics Support Force, Harbin, Heilongjiang, China.

The original version of this article has been updated.

