# Supplementary material for: Molecular typing of stage IB non-small-cell lung cancer for precision medicine
Source: Front Oncol. 2026 Mar 13;16:1605054. doi: 10.3389/fonc.2026.1605054 (PMC13021416; doi:10.3389/fonc.2026.1605054)
Supplement: Supplementary file 1 [file DataSheet1.pdf]

Figure S1

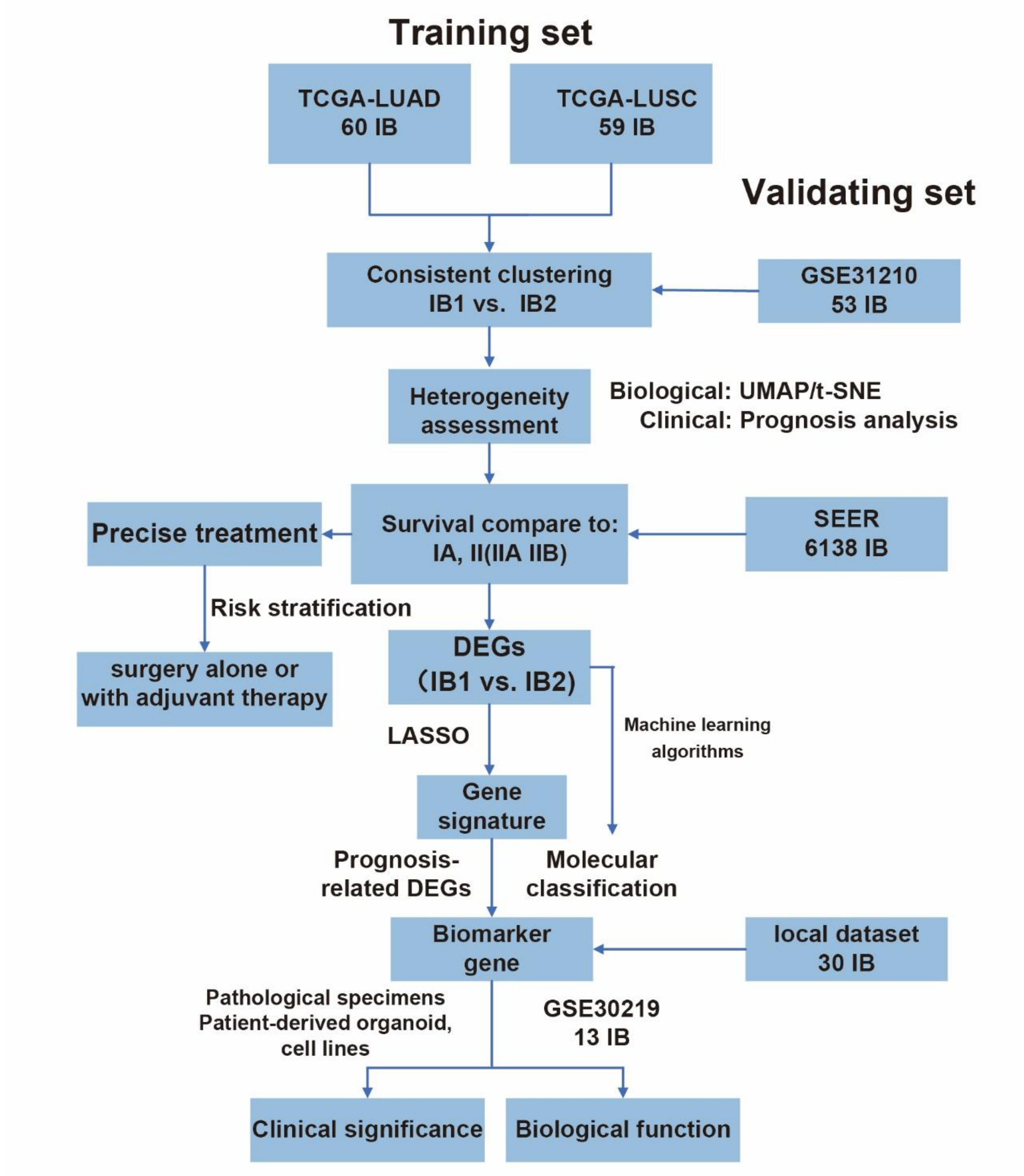

Figure S1. Study design

Figure S2

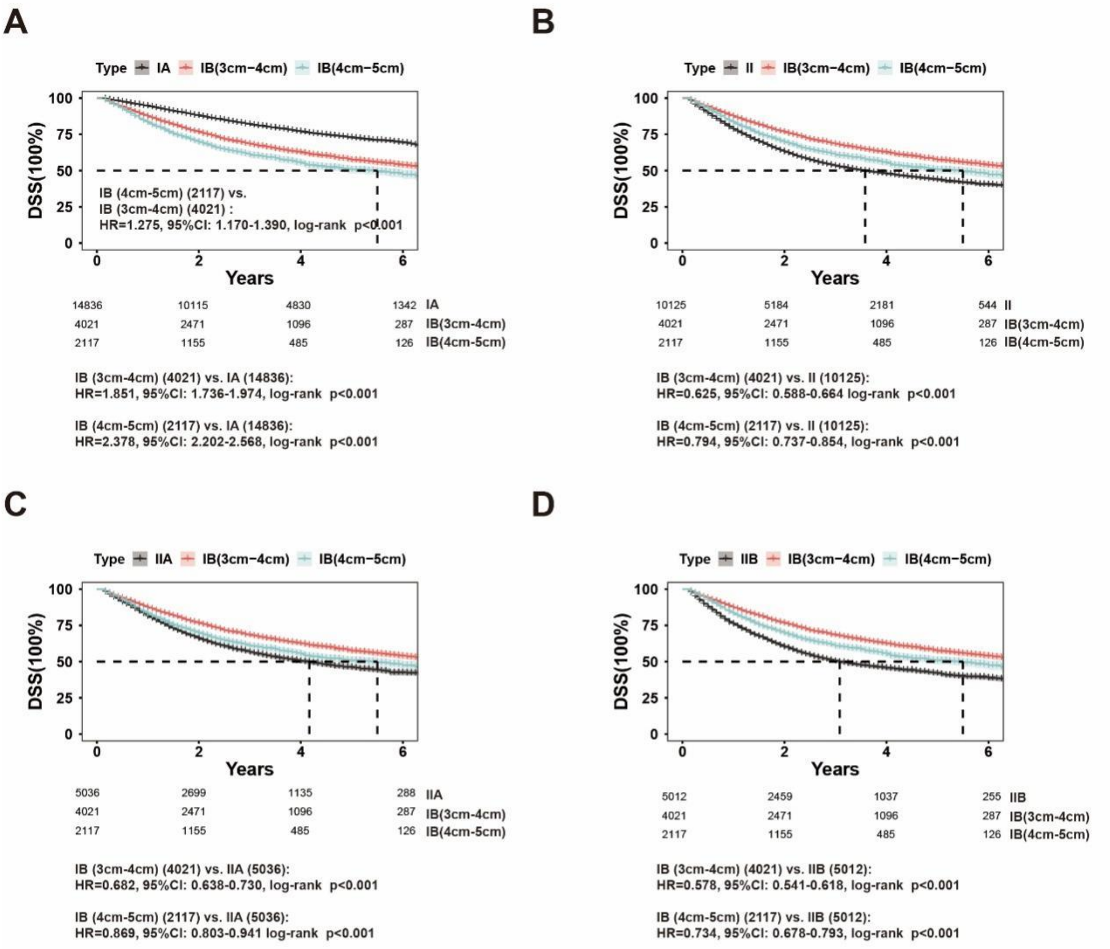

Figure S2. Evaluation of the clinical significance of stage IB NSCLC in the AJCC 8th edition system. DSS assessment between stage IB (3 cm-4 cm)/(4 cm-5 cm) patients and stage IA patients (A), stage II patients (B), stage IIA patients (C), and stage IIB (D) patients.

Figure S3

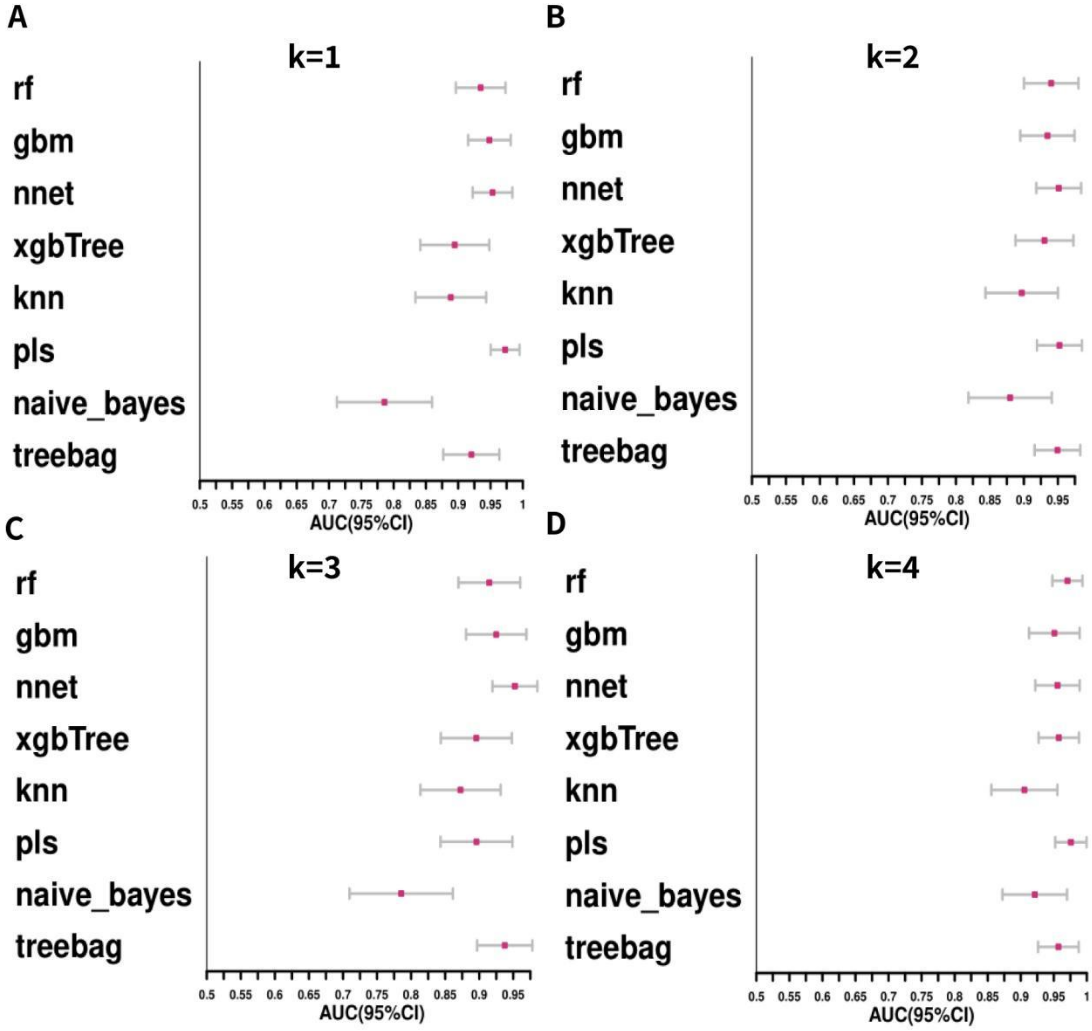

Figure S3. K-fold cross-validation results of the machine learning models.

Figure S4

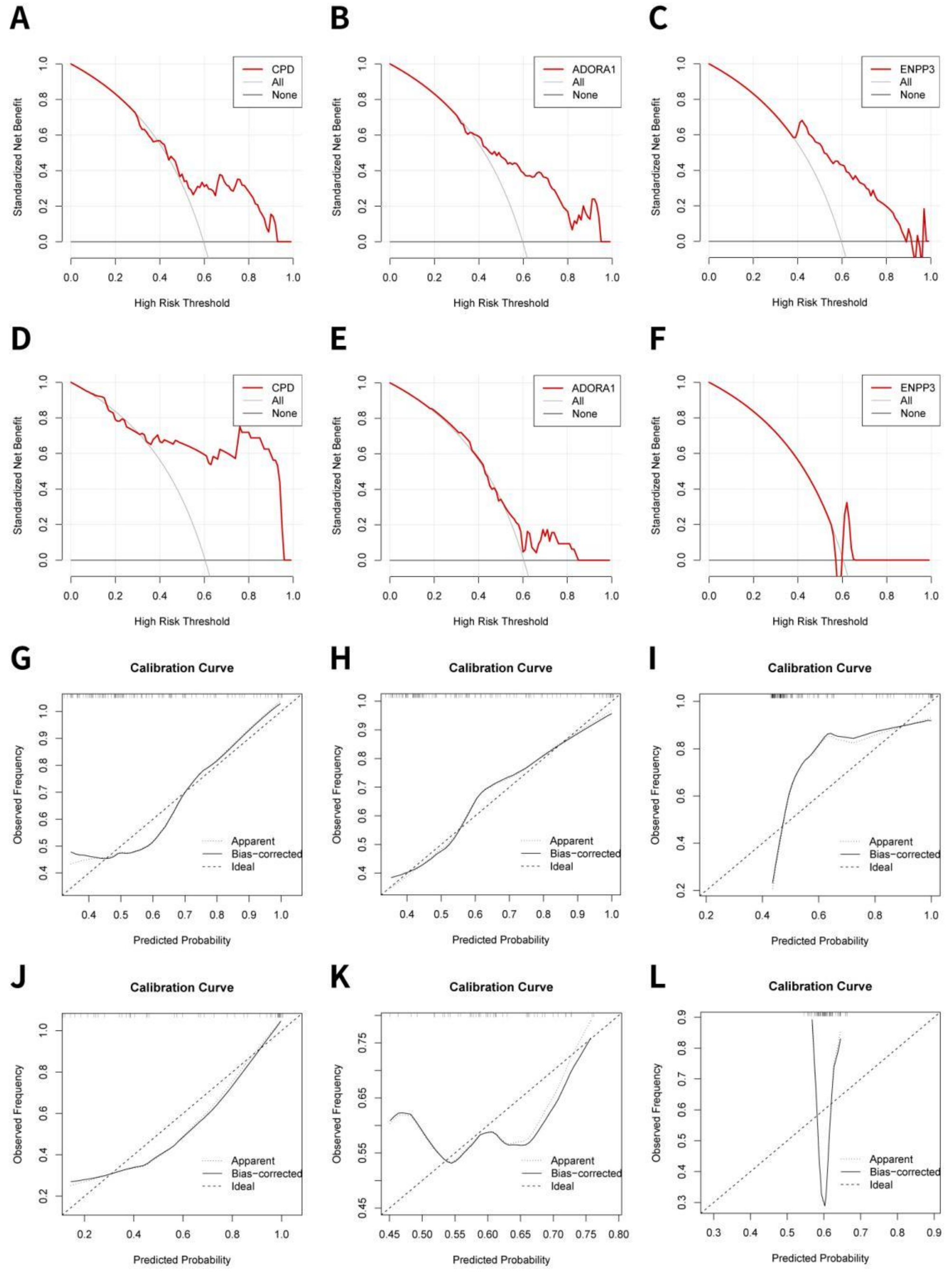

Figure S4. Decision Curve Analysis (DCA) and Calibration Curves for the three candidate gene signatures (CPD, ADORA1, and ENPP3). DCA of CPD (A), ADORA1 (B), and ENPP3 (C) in the TCGA training set. DCA of CPD (D), ADORA1 (E), and ENPP3 (F) in the GSE31210 validation set. Calibration curves of CPD (G), ADORA1 (H), and ENPP3 (I) in the TCGA training set. Calibration curves of CPD (J), ADORA1 (K), and ENPP3 (L) in the GSE31210 validation set.

Figure S5

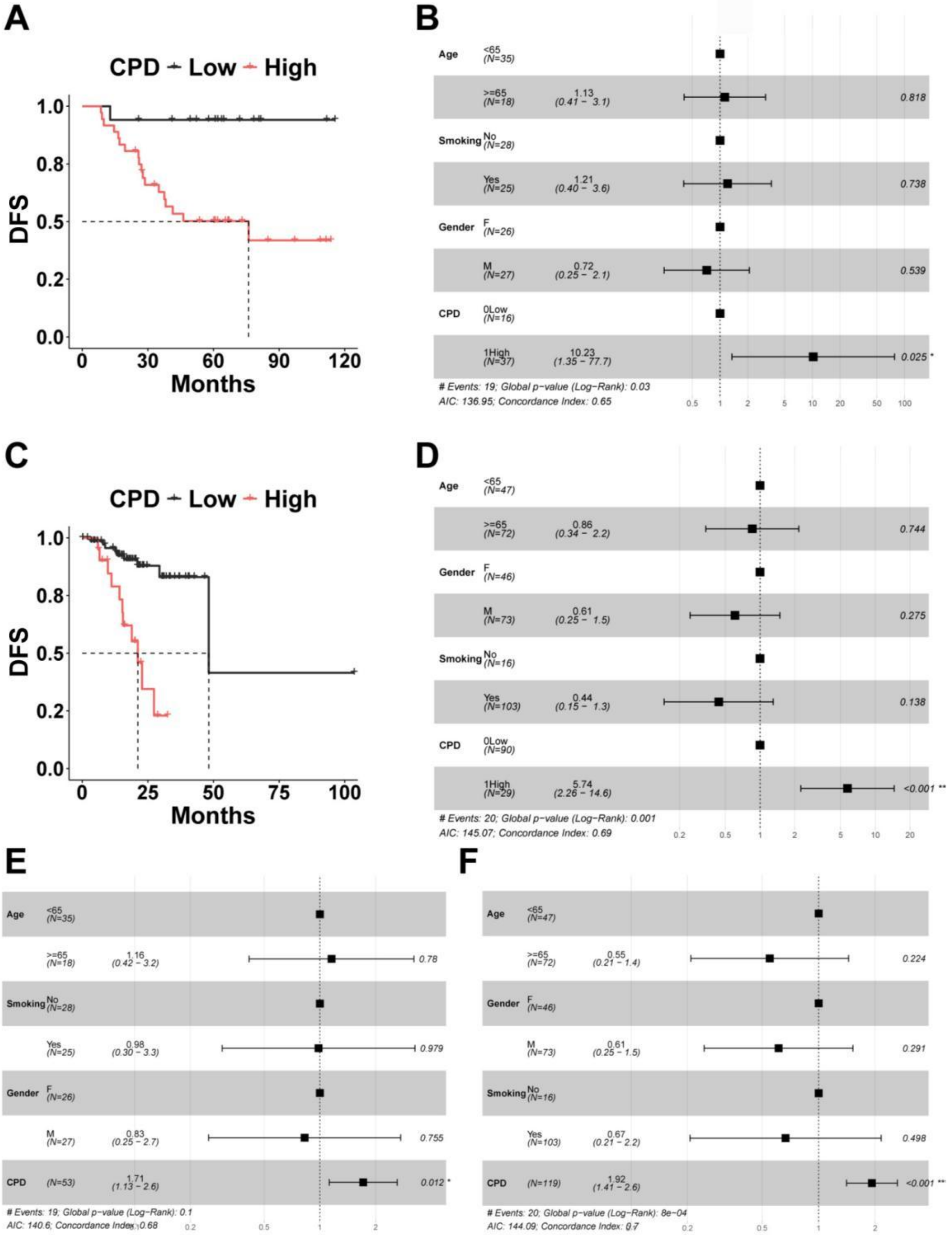

Figure S5. Validation of the prognostic value of CPD by two statistical approaches. Assessment based on data-driven optimal cut-points, showing DFS curves and multivariate analysis in GSE31210 (A-B) and TCGA (C-D). Assessment treating CPD as a continuous variable, showing multivariate analysis in GSE31210 (E) and TCGA (F).

Figure S6

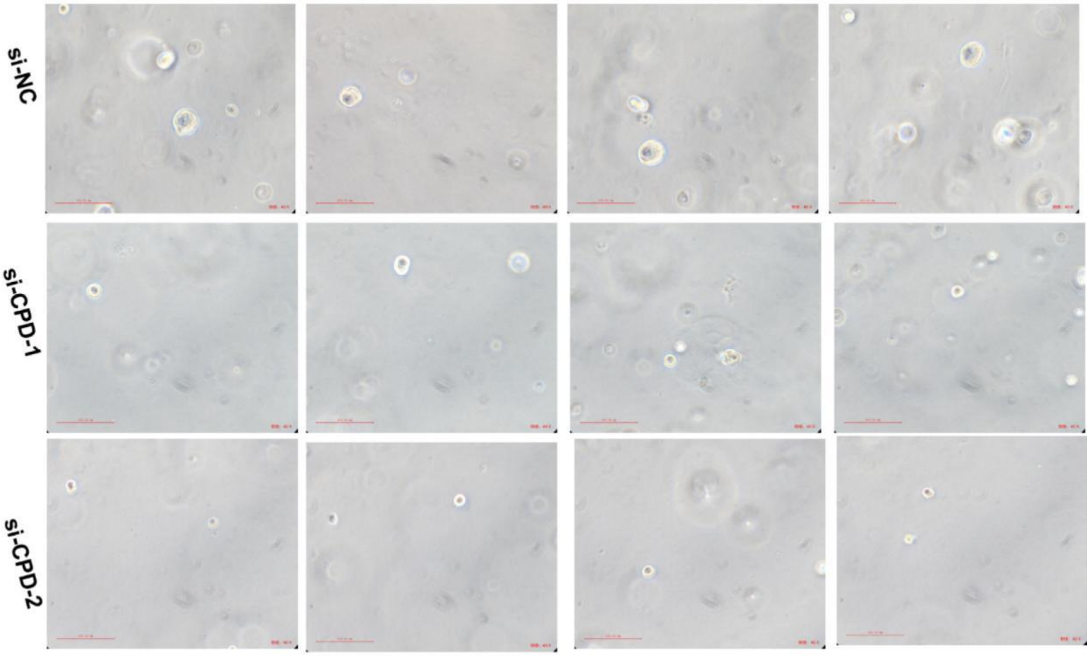

Figure S6. Knockdown of CPD inhibits tumor growth in patient-derived organoids (PDOs) of stage IB NSCLC

Figure S7

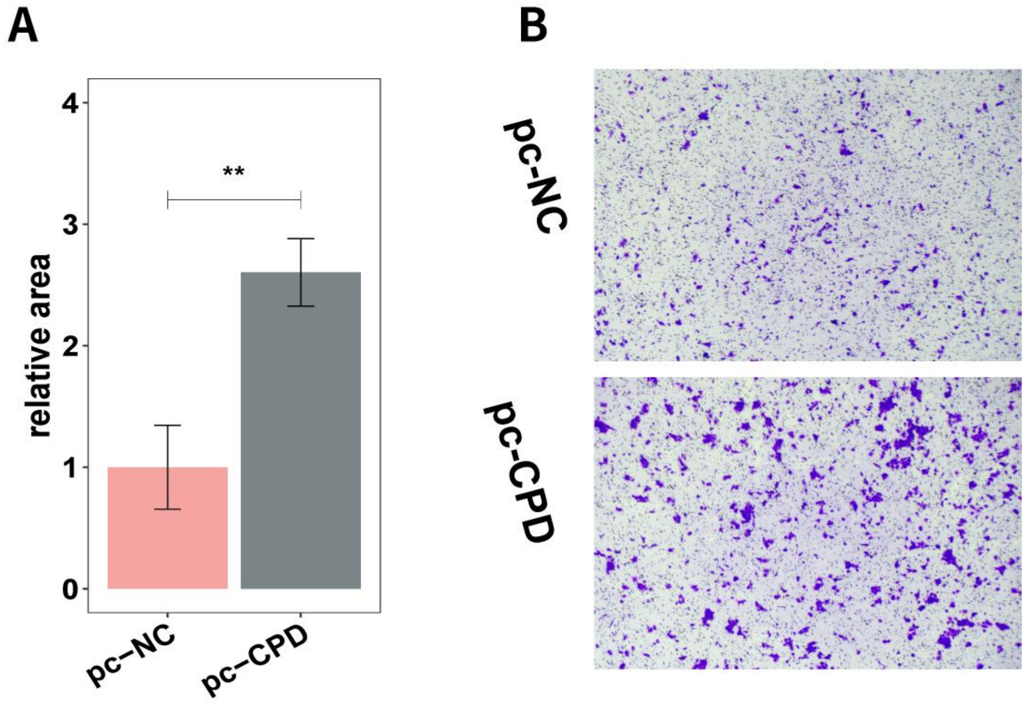

.

Figure S7. CPD promotes cell migration in the H358 cells. (A) Quantitative analysis of migrated cells. (B) Representative images of Transwell migration assays.
